# Intraoperative Bleeding in Pediatric Otolaryngology: Trends and Future Perspectives

**DOI:** 10.3390/pediatric15040063

**Published:** 2023-11-20

**Authors:** Antonino Maniaci, Salvatore Cocuzza, Ron B. Mitchell, Ignazio La Mantia, Luigi La Via

**Affiliations:** 1 Faculty of Medicine and Surgery, University of Enna Kore, 94100 Enna, Italy; 2 Department of Medical, Surgical Sciences and Advanced Technologies “GF Ingrassia” ENT Section, University of Catania, 95123 Catania, Italy; s.cocuzza@unict.it (S.C.); igolama@gmail.com (I.L.M.); 3 UT Southwestern Medical Center, Dallas, 75001 TX, USA; ron.mitchell@utsouthwestern.edu; 4 Department of Anaesthesia and Intensive Care, Univeristy Hospital Policlinico-San Marco, 24046 Catania, Italy; luigilavia7@gmail.com

## 1. Introduction

A significant challenge that ENT surgeons often encounter is managing intraoperative bleeding, a task that requires precision, adept judgment, and a thorough knowledge of the latest techniques and procedures [1].

**Figure 1:**
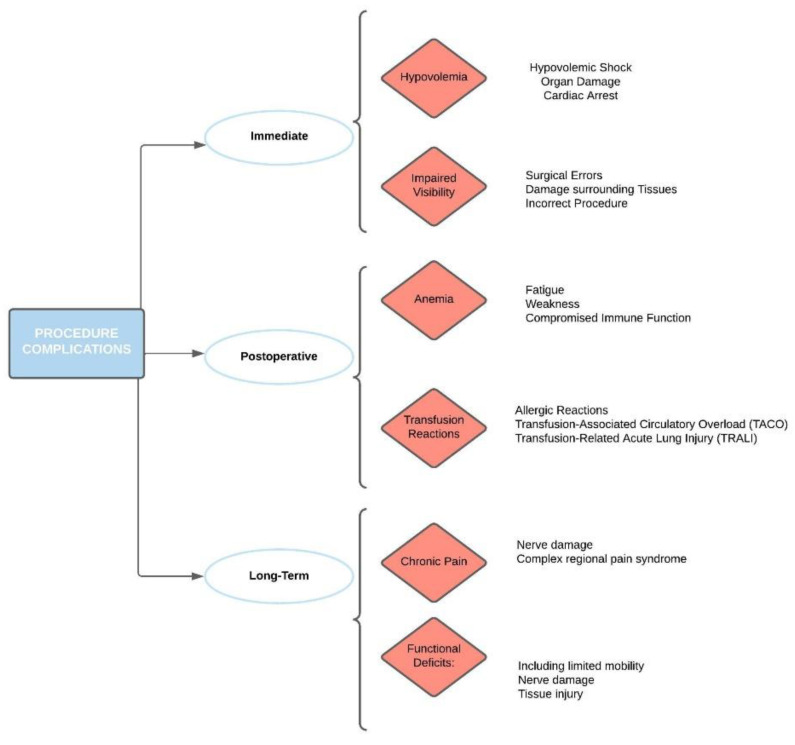
Procedure-related complications and relative comorbidities.

Intraoperative bleeding management is critical as it directly impacts surgical visualization, the duration of the procedure, and, most importantly, patient safety. When it comes to pediatric patients, these factors take on even greater significance due to the physiological differences and lower blood volume compared to adults, which make children more susceptible to the adverse effects of blood loss [2]. Pediatric patients present unique challenges in terms of blood volume and hemodynamic stability. Given their smaller blood volumes, children are potentially more susceptible to the effects of significant blood loss, which can rapidly lead to hemodynamic instability and hypovolemic shock [3] (Figure 1). 

Excessive bleeding obscures the surgical field, rendering it more difficult for the surgeon to navigate and potentially increases the risk of inadvertent damage to surrounding structures. It is worth noting that the sources of bleeding during otolaryngological procedures can vary widely, from larger vessels that can be directly sutured or clamped, to diffuse capillary oozing from the mucosa that might be more appropriately managed with topical hemostatic agents or cautery. 

### Therapeutic Management

Several strategies are often employed to manage intraoperative bleeding in pediatric otolaryngology surgeries. These might include the use of electrocautery, laser technology, radiofrequency ablation, and ultrasonic energy devices for their precision and ability to minimize tissue damage (Table 1). 

Each of these techniques possesses its strengths and limitations, and the choice often depends on the surgeon’s preference, the nature of the procedure, and the specific patient characteristics.

Electrocautery, for instance, is commonly used due to its accessibility and effectiveness in achieving hemostasis. However, it also carries risks, such as thermal injury to surrounding tissues. Laser technology provides excellent precision but requires significant safety precautions to prevent eye injuries and fire hazards [4]. However, each of these methods has potential drawbacks and risks, including thermal injury to surrounding tissues, allergic reactions, and, in the case of mechanical methods, potential damage to nearby structures.

Given the rapid advancements in technology, it is essential for surgeons to stay abreast of the latest research and developments. Formal training and continuing education in the use of these technologies are integral components of improving patient outcomes and reducing intraoperative complications.

In addition to advancements in surgical techniques and tools, effective communication between the surgical team, anesthesia providers, and nursing staff is vital for optimal intraoperative bleeding management. A well-coordinated team can promptly respond to changes in the patient’s status, making necessary adjustments to the surgical strategy and ensuring the child’s safety.

Additionally, topical hemostatic agents and surgical hemostats, such as sutures, clips, and staples, can be used to control bleeding [5]. Therefore, the choice of method often relies on the specific surgical context, the surgeon’s expertise, and the characteristics of the patient. Furthermore, the prevention of bleeding is equally as important as its management. This might involve careful preoperative planning, including assessing the patient’s coagulation status, optimizing their health prior to surgery, and meticulous surgical techniques to minimize tissue trauma.

Looking forward, the management of intraoperative bleeding in pediatric otolaryngology is a multifaceted challenge that requires a comprehensive approach tailored to the unique needs of pediatric patients, the characteristics of the surgical area, and the specific circumstances of each case. The combination of emerging technologies, evidence-based practices, ongoing training, and efficient teamwork holds the key to enhancing patient outcomes.

Research initiatives and comparative studies that focus on assessing the effectiveness and safety of different intraoperative bleeding management techniques in pediatric populations are needed. In addition, quality improvement projects focused on this topic could provide valuable insights into best practices and areas of potential improvement.

## Figures and Tables

**Table 1:** Different techniques described in literature for intraoperative bleeding.

Method	Description	Advantages	Disadvantages
Electrocautery	Uses electric current to coagulate tissue and stop bleeding.	Widely available and effective in achieving hemostasis.	Risk of thermal injury to surrounding tissues, smoke production.
Laser Technology	Uses focused light to cut or coagulate tissue.	High precision, minimal tissue damage.	Requires safety precautions to prevent eye injury and fire, expensive.
Radiofrequency Ablation	Uses radiofrequency energy to coagulate tissue.	Minimal tissue damage and less smoke production compared to electrocautery.	Can be slower than other methods, expensive.
Ultrasonic Energy Devices	Uses ultrasonic vibrations to cut and coagulate tissue.	Fast, minimal tissue damage, no smoke production.	Potential for unintended tissue damage if not used properly, expensive.
Topical Hemostatic Agents	These include gels, foams, and bandages that promote clotting.	Can be used in conjunction with other methods, useful for minor bleeding.	Not effective for severe bleeding, potential risk of allergic reaction.
Surgical Hemostats	These include sutures, clips, and staples used to control bleeding mechanically.	Direct control of bleeding vessels, permanent.	Requires access to the bleeding vessel, potential for tissue damage.

## Data Availability

No new data were created or analyzed in this study. Data sharing is not applicable to this article.
